# Pituitary adenoma and intracerebral aneurysms: case series, systematic review and meta-analysis

**DOI:** 10.1007/s11102-026-01690-w

**Published:** 2026-05-16

**Authors:** Valentino Marino Picciola, Michela Borghesi, Vanessa Trombin, Serena Chirico, Maria Rosaria Ambrosio, Maria Chiara Zatelli

**Affiliations:** 1 Section of Endocrinology, Geriatrics and Internal Medicine, Department of Medical Sciences, University of Ferrara, Via Ariosto 35, Ferrara, 44124 Ferrara Italy; 2 Department of Economics and Management, University of Ferrara, Ferrara, Italy; 3 Endocrine Unit, University Hospital S. Anna, Ferrara, 44124 Italy

**Keywords:** Pituitary Adenoma, Aneurysm, Prolactinoma, Acromegaly, PitNET, metanalysis

## Abstract

**Purpose:**

Intracranial aneurysms (IAs) and Pituitary Adenoma (PA) coexistence has been reported for decades, yet available evidence remains fragmented. We performed a systematic review and meta-analysis following PRISMA 2020 guidelines to assess clinical features of patients with both conditions and quantify the prevalence of IAs in PA patients.

**Methods:**

PubMed and Embase were searched up to December 15, 2025. Studies reporting coexistence of IAs and PAs were included. Individual patient-level data (Group 1) and aggregate prevalence data (Group 2) were analyzed separately. Risk of bias (RoB) was assessed using Joanna Briggs Institute tools. All analyses were performed using *metaprop* function from the *meta* package in R software.

**Results:**

Seventy-one studies met inclusion criteria. Most studies have a low RoB. In Group 1 (74 patients), PAs were predominantly macroadenomas (91.9%), most frequently non-functioning (40.5%). IAs were mainly in the cavernous segment of the internal carotid artery (33%). IA diagnosis occurred significantly later than PA diagnosis (*p* < 0.01). In Group 2 (5264 patients), the pooled prevalence of IAs among PAs was 4% (95% CI 2–4%; I² = 92.1%, τ² = 1.1050, *p* < 0.0001). Sex-stratified prevalence was 8% (95% CI: 6%-9%) in females and 6% (95% CI: 4%–7%) in males. IAs sex-specific prevalence was significantly higher in both male and female PA patients as compared to the general population (*p* < 0.01).

**Conclusion:**

Patients with PAs show a higher IAs prevalence compared with the general population, likely reflecting combined hemodynamic, anatomical, and endocrine influences.

**Supplementary Information:**

The online version contains supplementary material available at 10.1007/s11102-026-01690-w.

## Introduction

The association between intracranial aneurysms (IAs) and Pituitary Adenoma (PA) has long been recognized [1–71]. IAs are observed alongside primary brain tumors, with reported prevalence of 0.5–7.4%. Among the tumors associated with IA, PAs are the most common [64, 72]. This association may reflect complex interactions between anatomical, hemodynamic, and endocrine factors. Proposed mechanisms include direct mechanical effects of tumor expansion on adjacent vessels, local alterations in blood flow dynamics, hormonal or paracrine influences on vascular remodeling, and iatrogenic factors related to surgical, medical or radiotherapeutic treatment [1–71]. Several critical gaps persist in the existing literature concerning the clinical relevance of this association for preoperative planning, surgical risk stratification, and long-term patient management. First, the available evidence is largely derived from case reports, small case series, and heterogeneous, predominantly retrospective observational studies, making it difficult to draw reliable conclusions about the prevalence of IAs in patients with PAs. Second, no prior systematic review with meta-analysis has comprehensively synthesized the available evidence to quantify the pooled prevalence of this association or to characterize the demographic and clinical features of patients harboring both conditions. To address these gaps, the present systematic review and meta-analysis aims to: (1) synthesize the available evidence on the clinical characteristics of patients with coexisting IAs and PAs; (2) quantify the pooled prevalence of IAs in patients with PAs; and (3) identify demographic, hormonal, and tumor-related factors that may influence this association. This study was conducted in accordance with the Preferred Reporting Items for Systematic Reviews and Meta-Analyses (PRISMA) 2020 guidelines [73].

## Materials and methods

### Search strategy

A comprehensive literature search was conducted in PubMed and Embase via Elsevier. The search strategy combined controlled vocabulary (MeSH in PubMed and Emtree in Embase) and free-text terms to maximize sensitivity. The strategy was adapted to the syntax and indexing system of each database. The complete search strings for each database are provided in Supplementary Table 1. No restrictions on study design or publication type were applied. A language filter was applied to include only articles published in English or Italian. The last search was performed on June 12, 2025, and updated on December 15, 2025, which was defined as the data lock date for study inclusion. Articles published after this date were not included. Duplicate records were identified and removed.

## Eligibility criteria

We included studies reporting cases or cohorts of coexisting IAs and PAs, with both conditions confirmed by imaging, without restrictions on patient age or study design. In studies reporting autopsy data, additional aneurysms identified at post-mortem examination were also included in the analysis, provided that the coexistence of at least one IA and the PA had been established by imaging during life. We excluded abstracts as well as reviews, editorials, and other publications not reporting original data. Studies were also excluded if they involved mycotic or marantic aneurysms, or pseudoaneurysms. Pseudoaneurysms included both traumatic and iatrogenic forms, the latter defined as aneurysms not detected on preoperative imaging but appearing immediately after neurosurgery. For the purposes of this review, studies reporting individual patient-level data were classified as Group 1, while studies reporting only aggregate prevalence data were classified as Group 2. For Group 1, studies were excluded if they did not report patient demographic characteristics (age, sex), IAs characteristics (number/patient, age at diagnosis, anatomical site), or PA characteristics (size, hormonal function). For Group 2, studies were excluded if they did not report either the total number of patients with PA or the number of patients with coexisting IAs.

## Study screening and data extraction

Articles were screened by title and abstract and then retrieved and evaluated according to the predefined inclusion and exclusion criteria. Additionally, the reference list of each included study was manually screened to identify any additional relevant publications. Data from the included studies were systematically collected using a standardized Microsoft Excel spreadsheet. All data were manually extracted. Extracted variables are presented in Supplementary Table 2. Two independent Authors performed all tasks. Discrepancies were resolved through discussion and consensus, with the involvement of a third reviewer when necessary.

## Risk of bias assessment (RoB)

RoB was assessed using approaches tailored to study design. For Group 1, we applied the Joanna Briggs Institute (JBI) Checklist for case reports [74] (Supplementary Table 3), and JBI Checklist for case series [75] (Supplementary Table 4). For Group 2, we used the JBI Checklist for Prevalence Studies [76] (Supplementary Table 5). RoB assessments were used to characterize the overall quality of the evidence, and no studies were excluded on the basis of RoB.

## Reporting bias

Publication bias was not formally assessed with funnel plots because, in meta-analyses of proportion studies with extreme outcomes, conventional plots may produce asymmetry due to statistical artifacts rather than true bias [77]. In addition, the small number and heterogeneity of included studies further limited the interpretability of funnel plot-based assessments [77].

### Cases series

We report 3 patients with IA and concomitant PAs, identified retrospectively from our clinical records at the Endocrinology Unit, Dept of Specialty Medicines of the Ferrara Hospital (Ferrara, Italy) between 1990 and 2025. Selection was not systematic and intended for descriptive purposes. However, given the rarity of the association, relevant data were included in Table 1 and in statistical analysis for Group 1.

**Table 1:** Included studies reporting individual patient-level (Group 1)

Studies		Patient	PA features			IAs features			Previously proposed risk factors for coexistence PitNETs and IAs			Apoplexy
Author, year	Type	Sex	Age at diagnosis	Size	Hormonal function	Number	Age at diagnosis	Site onset	Previous skull base surgery	Previous brain radiotherapy	Hypertension	
Acqui M. et al., 1987	Case series	M	48	Macro	GH-secreting	1	48	ACoA, left	No	No	NA	No
Acqui M. et al., 1987	Case series	F	27	Micro	GH-secreting	1	38	OA, left	No	No	NA	No
Adachi K. et al., 1993	Case report	F	45	Micro	PRL-secreting	1	45	ACoA, NA	No	No	NA	No
Agarwal H. et al., 2018	Case report	F	49	Macro	Non-functioning	2	49	Rt P-ICA, right P-ICA, left	No	No	NA	No
Akutsu N. et al., 2014	Case report	M	58	Macro	PRL-secreting	1	58	CS-ICA, left	No	No	NA	No
Almeida Silva JM et al., 2013	Case report	M	43	Macro	PRL-secreting	1	53	ACoA, right	Yes, 10 years before IA	No	NA	No
Bulsara K. et al., 2007	Case report	M	73	Macro	Non-functioning	1	73	ACoA, NA	No	No	NA	No
Choi HS.et al., 2013	Case report	F	70	Macro	Non-functioning	1	70	SHA, right	No	No	NA	No
Chuang C.C et al., 2006	Case report	M	60	Macro	Non-functioning	1	60	CS-ICA, left	No	No	NA	Yes
Curto L. et al., 2007	Case report	F	48	Macro	GH-secreting	2	61 62	CS-ICA right CS-ICA, left	Yes, 13 years before IA	No	NA	No
Fedder S. L. et al., 1990	Case report	F	68	Macro	Non-functioning	1	66	ACoA, left	No	No	NA	No
Fujiwara S. et al., 1991	Case series	F	47	Macro	GH-secreting	1	47	ICA-OA, left	No	No	NA	No
Fujiwara S. et al., 1991	Case series	M	59	Macro	PRL-secreting	1	59	ICA-PCA, left	No	No	NA	No
Fujiwara S. et al., 1991	Case series	F	53	Macro	Non-functioning	1	69	ICA-PCA, right	Yes, 13 years before IA	Yes, 13 years before IA	NA	No
Hermier M. et al., 1994	Case report	M	58	Macro	FSH/alpha-subunit secreting	1	58	ACoA	No	No	NA	No
Hori T. et al., 1982	Case report	F	45	Macro	GH-secreting	1	60	ICA, right	No	No	NA	No
Imamura J et al.	Case report	F	72	Macro	PRL-secreting	1	72	CS-ICA, right	No	No	NA	No
Inoue H. et al., 2019	Case report	M	41	Macro	GH-secreting	2	61	CS-ICA, right CS-ICA, right	Yes, 20 years before IA	Yes, 20 years before IA	NA	No
Jordan RM et al.	Case report	M	51	Macro	GH-secreting	1	51	ACoA, right	No	No	NA	No
Khachatryan T. et al., 2018	Case report	F	37	Macro	GH-secreting	1	37	CS-ICA, right	No	No	NA	No
Khalsa S. S. et al., 2016	Case report	M	61	Macro	PRL-secreting	1	61	CS-ICA, right	No	No	NA	No
Kino H et al.	Case report	F	53	Macro	Non-functioning	1	53	S-ICA, right	No	No	NA	No
Lippman H. H. et al., 1971	Case report	M	50	Macro	Non-functioning	2	50	CS-ICA, left I-ICA, right	No	No	NA	No
Mangiardi J. R. et al., 1983	Case report	M	47	Macro	Non-functioning	2	47 56	NA S-ICA, NA	Yes, 9 years before IA	No	NA	No
Nishijima Y. et al., 2010	Case report	F	40	Micro	ACTH-secreting	1	40	CS-ICA, right	No	No	NA	No
Revuelta R. et al., 2002	Case report	F	60	Macro	Non-functioning	1	60	S-ICA, left	No	No	NA	No
Sade B. et al., 2004	Case report	F	39	Macro	GH-secreting	1	39	CS-ICA, left	No	No	NA	No
Salpietro F. M. et al., 1997	Case report	F	71	Macro	Non-functioning	1	71	CS-ICA, left	No	No	Yes	No
Satyarthee G. D. & Raheja A., 2017	Case report	M	52	Macro	GH-secreting	1	52	S-ICA, right	No	No	NA	No
Schenk V. W. & Solleveld H., 1968	Case report	M	40	Macro	GH-secreting	5	56 57 Post-mortem Post-mortem Post-mortem	MCA, right MCA, left Pons Varolii Cerebellum Thalamus	No	Yes, 10 and 16 years before IA	NA	No
Seda L. Jr et al., 2008	Case report	F	58	Micro	GH-secreting	1	58	S-ICA, left	No	No	Yes	No
Shahlaie K. et al., 2005	Case report	F	46	Macro	PRL-secreting	1	46	ACoA, left	No	No	NA	Yes
Soni A et al.	Case report	M	53	Macro	PRL-secreting	1	53	CS-ICA, right	No	No	NA	Yes
Tian X et al.	Case report	F	63	Macro	PRL-secreting	1	63	ACoA, right	Yes, 6 months before IA	No	NA	No
Troisi F et al.	Case report	M	48	Macro	GH-secreting	1	48	ACoA, left	No	No	NA	No
Wakai S. et al., 1979	Case series	F	68	Macro	Non-functioning	1	68	PCoA, left	No	No	NA	No
Wakai S. et al., 1979	Case series	F	34	Macro	PRL-secreting	1	34	OA, left	No	No	NA	No
Wakai S. et al., 1979	Case series	F	36	Macro	PRL-secreting	1	36	PCoA, right	No	No	NA	No
Wakai S. et al., 1979	Case series	M	66	Macro	Non-functioning	2	66	ACA (A1), right	No	No	NA	No
Wakai S. et al., 1979	Case series	M	49	Macro	GH-secreting	1	49	CS-ICA, left	No	No	Yes	No
Wang CS et al., 2009	Case report	F	61	Macro	PRL-secreting	1	61	S-ICA, NA	No	No	Yes	No
Xia X. et al., 2011	Case report	F	48	Macro	GH-secreting	1	48	CS-ICA, left	No	No	NA	No
Xu K. et al., 2015	Case report	M	49	Macro	PRL-secreting	1	49	ACoA, left	No	No	NA	Yes
Yamada S. et al., 2012	Case report	F	57	Macro	Non-functioning	1	57	S-ICA, left	No	No	NA	No
Yang MY. et al., 2005	Case report	F	53	Macro	PRL-secreting	1	53	CS-ICA, left	No	No	NA	No
Yu K. et al., 2011	Case report	F	54	Macro	Non-functioning	1	54	SHA, left	No	No	NA	No
Zatelli M. C. et al., 2004	Case report	M	58	Micro	GH-secreting	1	58	MCA, right	No	No	Yes	No
Cifuentes-Lobelo H. et al., 2024	Case report	F	71	Macro	GH-secreting	1	71	ACoA, left	No	No	Yes	No
He W. et al., 2024	Case report	M	55	Macro	Non-functioning	1	55	ACoA, left	No	No	NA	No
Fookeerah P. & McLean M., 2023	Case report	M	41	Macro	GH-secreting	1	65	ACA, left	Yes, 24 years before IA	Yes, 24 years before IA	Yes	No
Gu Y. et al., 2022	Case report	F	51	Macro	Non-functioning	1	51	P-ICA, NA	No	No	NA	No
Mondragon-Soto M. G. et al., 2022	Case report	F	55	Macro	Non-functioning	2	55 55	P-ICA, right P-ICA, left	No	No	NA	No
Holdaway M. et al., 2023	Case report	M	81	Macro	Non-functioning	1	81	CS-ICA, right	No	No	NA	No
Yamashita S. et al., 2023	Case series	M	37	Macro	PRL-secreting	1	54	CS-ICA, right	No	Yes, 16 years before IA	No	No
Yamashita S. et al., 2023	Case series	F	45	Macro	PRL-secreting	1	60	Rt CS-ICA, right	No	Yes, 14 years before IA	Yes	No
Yamashita S. et al., 2023	Case series	F	46	Macro	GH-secreting	1	68	Rt CS-ICA, right	No	No	NA	No
Rennert R. C et al., 2022	Case report	F	35	Micro	ACTH-secreting	1	35	P-ICA, left	No	No	NA	No
Nene A. et al., 2022	Case report	F	66	Macro	Non-functioning	1	66	PARA-ICA, NA	No	No	NA	No
Yoshida M. et al., 2021	Case report	M	78	Macro	Non-functioning	1	78	ACA (A1), right	No	No	NA	Yes
Wang T et al., 2021	Case report	F	38	Macro	Non-functioning	1	38	CS-ICA, right	No	No	NA	No
Taniguchi T. et al., 2021	Case report	F	53	Macro	Non-functioning	1	65	CS-ICA, left	Yes, 12 years before IA	Yes, 12 years before IA	NA	No
Pedrozo C. A. et al., 2020	Case report	M	55	Macro	Non-functioning	1	55	CS-ICA, left	No	No	Yes	Yes
Nakahara M et al., 2018	Case report	F	27	Macro	PRL-secreting	1	61	CS-ICA, left	No	No	NA	No
McConachie N. S. & Jacobson I.	Case report	F	34	Macro	GH-secreting	2	51 51	CS-ICA, right CS-ICA, left	No	Yes, 17 years before IA	NA	No
Gokalp H. Z. et al.,	Case series	M	45	Macro	Non-functioning	1	45	ACoA, right	No	No	NA	Yes
Keenan J. P. et al., 2021	Case report	M	67	Macro	Non-functioning	2	67 67	CS-ICA, left ACoA, left	No	No	NA	No
Giammusso V., 1960	Case report	F	58	Macro	GH-secreting	1	58	CS-ICA, right	No	No	Yes	No
Villalobos-Diaz R., 2024	Retrospective	F	55	Macro	GH-secreting	1	55	P-ICA, left	No	No	NA	No
Taiki S. et al., 2017	Case report	F	73	Macro	Non-functioning	1	73	P-ICA, right	No	No	NA	No
Hidenori E. et al., 2011	Case report	F	43	Macro	PRL-secreting	1	64	Petrosus ICA, left	Yes, 19 years before IA	Yes, 19 years before IA	NA	No
Nadjem H. et al., 2007	Case report	M	39	Macro	Non-functioning	1	42	BA, right	No	No	NA	No
Case 1, Marino Picciola et al., 2026		F	55	Macro	Non-functioning	1	55	MCA, left	No	No	Yes	No
Case 2, Marino Picciola et al., 2026		F	52	Macro	Non-functioning	1	52	S-ICA, left	No	No	No	No
Case 3, Marino Picciola et al., 2026		M	56	Macro	GH-secreting	2	70 70	OA, right OA, right	Yes, 25 years before IA	No	No	No

The table includes study characteristics, patient demographics, PA features (size and hormonal activity), IA characteristics (number, site, and age at diagnosis), and previously reported risk factors, including prior skull base surgery, radiotherapy, hypertension. Pituitary apoplexy was also recorded, given its potential relevance in this specific population

Macro = > 1 cm; Micro = < 1 cm, *M*  Male, *F*  Female, *NA* not available, *ICA* internal carotid artery, *CS-ICA* cavernous sinus, *ACoA* anterior communicating artery, *S-ICA* supraclinoid ICA, *P-ICA* paraclinoid ICA, *MCA* middle cerebral artery, *OA* ophthalmic artery, *ACA* anterior cerebral artery, *PCA* posterior cerebral artery, *PCoA* posterior communicating artery, *SHA* superior hypophyseal artery, *I-ICA* infraclinoid ICA, *BA* basilar artery, *N/A* not available, *PARA-ICA* paraophthalmic region, Petrosus-ICA, *P-Varolii* Pons Varolii

## Statistics

For both Group 1 and 2, categorical variables were summarized as absolute frequencies (*n*) and percentages (%), where percentages were calculated using as denominator the total number of subjects with available data for the variable of interest (complete-case analysis). For lesion subtype analyses, the prevalence of Macroadenoma and Microadenoma was estimated as the proportion of subjects presenting each lesion type (numerator = number of subjects with Macroadenoma or Microadenoma; denominator = total number of subjects evaluated within the relevant subgroup, e.g. men or women). Exact binomial 95% confidence intervals (95% CI) were calculated for all prevalence estimates. When subgroup analyses were performed to compare the proportion of Macroadenoma and Microadenoma lesions between men and women, a formal hypothesis test was conducted. The null hypothesis assumed equality of proportions between groups:

$$\:\left\{\begin{array}{c}{H}_{0}:{p}_{\mathrm{m}\mathrm{e}\mathrm{n}}={p}_{\mathrm{w}\mathrm{o}\mathrm{m}\mathrm{e}\mathrm{n}}\\\:{H}_{1}:{p}_{\mathrm{m}\mathrm{e}\mathrm{n}}\ne\:{p}_{\mathrm{w}\mathrm{o}\mathrm{m}\mathrm{e}\mathrm{n}}\end{array}\right.$$

Differences in proportions were assessed using the chi-square test of independence. When expected cell counts were less than five in any contingency table cell, Fisher’s exact test was applied instead. For these comparisons, effect sizes were expressed as absolute differences in proportions and corresponding 95% confidence intervals. All statistical tests were two-sided, and a $\:p$-value < 0.05 was considered statistically significant. Furthermore, to assess the temporal relationship in age at diagnosis of PA and IA, the non-parametric Wilcoxon signed-rank test was applied, as it is suitable for paired, non-normally distributed data. Finally, one-sample proportion tests were performed to compare the observed proportions in our sample with the corresponding proportions reported in the reference population [78], using the observed study prevalence as the sample estimate (numerator = number of cases observed in our cohort; denominator = total number of evaluable subjects) and the published proportion as the reference value under the null hypothesis. Forest plots were generated using the meta package in R to graphically display prevalence estimates and corresponding 95% confidence intervals. Where pooled prevalence estimates were derived, proportions were calculated using inverse-variance weighting, and between-study heterogeneity was assessed using Cochran’s Q statistic and the I² statistic. All analyses were performed using the *metaprop* function from the *meta* package in R software (R Foundation for Statistical Computing, Vienna, Austria) (Supplementary Table 6).

## Results

### Studies selection

**Fig. 1:**
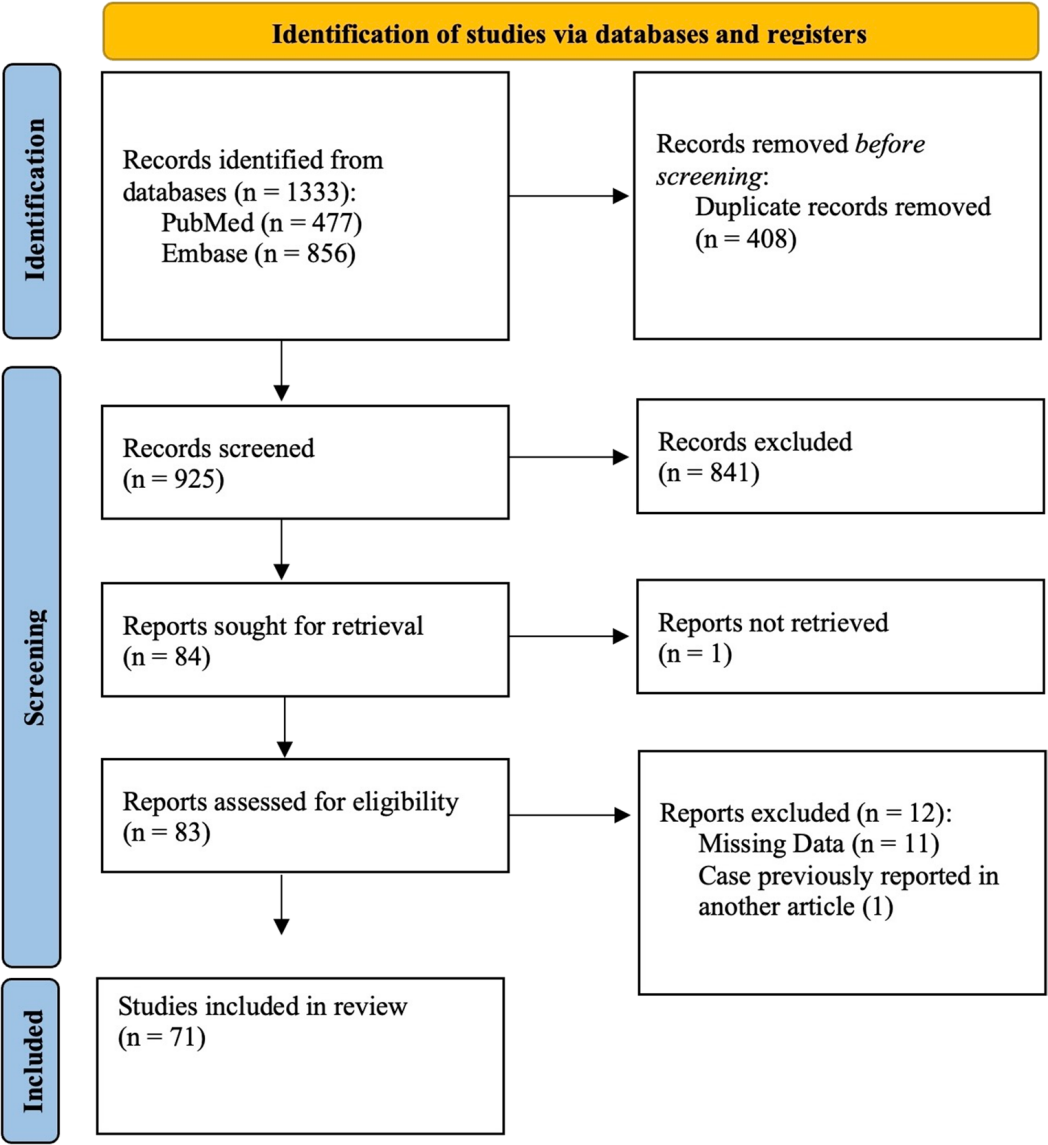
Title: PRISMA 2020 flow diagram for present systematic review Caption: Flow diagram illustrating the process of study identification, screening, eligibility assessment, and inclusion according to PRISMA 2020 guidelines. A total of 1,333 records were identified through database searching (PubMed: *n* = 477; Embase: *n* = 856). After removal of 408 duplicate records, 925 studies were screened by title and abstract, of which 841were excluded. Eighty-four reports were articles for retrieval, with 83 successfully assessed for eligibility. Twelve studies were excluded at the full-text stage (11 due to missing data and 1 due to duplicate reporting of the same case). A manual search of the reference lists of the included studies identified one additional eligible study (Shahlaie K. et al., 2005), which was included in the final count. Ultimately, 71 studies were included in the systematic review and meta-analysis

A total of 1333 records were identified through database searches, of which 71 met the inclusion criteria and were included in the statistical analysis. The selection process is summarized in Fig. 1, while the characteristics of studies included in Group 1 and Group 2 are presented in Tables 1 and 2, respectively.

**Table 2:** Included studies reporting aggregate data (Group 2)

Studies			Patient with PA				Patient both with IAs and PA				
Author, year	Type	Total		F	M	Total		F	M	Multiple IAs	
Zheng H. et al., 2024	Retrospective	446		223	223	29		22	7	8	
Villalobos-Diaz R. et al., 2024	Retrospective	1666		NA	NA	5		4	1	1	
Xin M. et al., 2023	Retrospective	18		NA	NA	3		3	0	NA	
Jintao Hu, 2019	Retrospective	434		181	243	36		14	16	6	
Satoru O. et al., 2012	Retrospective	208		107	101	9		2	7	1	
Manara R. et al., 2011	Cross-sectional	152		82	70	26		19	7	10	
Pant B. et al., 1997	Retrospective	467		297	170	25		14	11	3	
Jakubowski J. 1978	Retrospective	150		66	84	10		4	6	0	
Min Chul O. et al., 2012	Retrospective	800		470	330	18		14	4	1	
Yoshikazu O. et al., 2022	Retrospective	923		NA	NA	24		14	10	4	

The table reports the total number of patients with PA and those with coexisting IA, including sex distribution and the occurrence of multiple IA, when available

*NA* Not Available, *F* Female, *M* Male

### Risk of bias

Overall, 72.4% of the included case reports were rated as providing high-quality evidence, 25.9% as moderate-quality evidence, and 1.7% as low-quality evidence (Supplementary Table 2). Among the included case series, 25% were rated as providing high-quality evidence, 50% as moderate-quality evidence, and 25% as low-quality evidence (Supplementary Table 3). Regarding prevalence studies, 50% were rated as providing high-quality evidence, 40% as moderate-quality evidence, and 10% as low-quality evidence (Supplementary Table 4).

### Case series

#### Case 1

A 55-year-old woman presented to the emergency department with seizures and head trauma. Her medical history included surgical correction of a patent ductus arteriosus in early adulthood, hysterectomy for uterine fibromatosis, hypertension, and hypertensive heart disease. She denied smoking and alcohol use. Brain CT and CT angiography revealed a large sellar mass with suprasellar and parasellar extension, erosion of the sellar floor, and subarachnoid hemorrhage caused by an 8 mm aneurysm of the left middle cerebral artery. The aneurysm was treated via a fronto-pterional approach with surgical clipping without complications. Subsequent endocrine evaluation revealed panhypopituitarism, and hormone replacement therapy was started. Several years later, imaging documented progression of the sellar lesion, leading to a transsphenoidal resection. Histopathology was consistent with a non-functioning (NF) PA. Postoperatively, permanent diabetes insipidus occurred. Follow-up imaging showed further tumor extension into the sphenoid sinus, right cavernous sinus, and suprasellar region approaching the third ventricle, prompting consideration for additional surgery.

#### Case 2

A 52-year-old woman with transfusion-dependent intermediate beta-thalassemia underwent a brain MRI for persistent headache. Her history included menarche at age 17, menopause at age 48 years managed with hormone replacement therapy, splenectomy, osteoporosis, previous rib fractures, hypercalciuria, mild hyperinsulinism and BMI = 16.7 kg/m^2^. She denied smoking. MRI revealed an intra- and suprasellar expansive lesion measuring 18 × 17 × 20 mm, along with a 4 mm saccular aneurysm of the left supraclinoid carotid artery and hypoplasia of the right anterior cerebral artery A1 segment, consistent with a normal anatomical variant. The lesions were confirmed by subsequent pituitary MRI and CT angiography. Hormonal work-up was unremarkable. The patient underwent transsphenoidal surgery without intra- or postoperative complications. Histopathology was consistent with the diagnosis of NF PA. Patient was disease free at the last follow-up.

#### Case 3

A 55-year-old man presented to the emergency department with diplopia for 1 month. Clinical history was notable for progressive enlargement of hands and feet, mild obstructive sleep apnea, obesity, and prior smoking. Brain MRI showed a sellar lesion with invasion of the sphenoid and bilateral cavernous sinuses. Endocrine evaluation was consistent with acromegaly. The patient underwent transsphenoidal surgery complicated by permanent diabetes insipidus and central hypogonadism. Histopathology was consistent with a GH-secreting PA. The patient was started on octreotide LAR therapy due to disease persistence. Several years later, a second transsphenoidal surgery was performed due to tumor recurrence. During long-term follow-up, he developed type 2 diabetes mellitus, osteoporosis, and a colon tubular adenoma with low-grade dysplasia. At age 70, angio-MRI follow-up demonstrated a 5 mm saccular aneurysm at the origin of the ophthalmic artery and a smaller 2 mm saccular aneurysm immediately below it. Given the small size of the aneurysms, a radiological follow-up strategy was adopted, and the lesions remained stable over time.

### Group 1 evaluation

#### Sex and age distribution

The study population comprised 74 patients, 59.5% females. We investigated the characteristics of patients with both PAs and IAs stratifying the population into five age groups: ≤39 years, 40–49 years, 50–59 years, 60–69 years, and ≥ 70 years. In this cohort, PA diagnosis occurred predominantly between 40 and 59 years of age, with a mean age at diagnosis of 52 years. The distribution followed a unimodal pattern centered in the fifth decade of life. A similar age distribution was observed for aneurysm occurrence, with the following percentages by age group: ≤39 years, 9.5%; 40–49 years, 17.6%; 50–59 years, 33.8%; 60–69 years, 27.0%; and ≥ 70 years, 12.2%. This distribution was likewise unimodal and centered in the fifth decade. The observed time interval between PA diagnosis and aneurysm ranged from 10 to 34 years. For each patient, the age at IA diagnosis was compared with the age at PA diagnosis. The median within-patient difference was positive, indicating that IA diagnosis occurred consistently later. This difference was statistically significant according to the Wilcoxon signed-rank test (*p* = 0.0003), confirming that IAs diagnosis occurred at a significantly later age.

### PAs features

In the collected dataset, 40.5% of PAs were NF, 31.1% GH-secreting, 24.3% PRL-secreting, 2.7% ACTH-secreting, and 1.4% gonadotropinoma. Macroadenomas accounted for 91.9% of cases. When stratified by sex, macroadenomas were observed in 29 of 30 men (96.7%) and in 39 of 44 women (88.6%). Although the proportion of macroadenomas was numerically higher in men, the difference between sexes was not statistically significant (Fisher’s exact test, *p* = 0.391).

### IAs features

Analysis of IA site is shown in Supplementary Fig. 1. Multiple IAs were identified in 13.5% of patients (*n* = 10), including 6 males and 4 females. Among these, 9 patients harbored two aneurysms, while 1 patient presented with five aneurysms.

### Risk factors

We assessed established risk factors for IA development, including hypertension and smoking. Data on blood pressure were available for 14 patients, of whom 11 were hypertensive. Smoking status was reported in 5 patients, 3 of whom were smokers. We also evaluated additional factors potentially influencing the development of PAs and IAs, such as previous cranial base surgery and brain radiotherapy. Eleven patients (15%) had undergone prior cranial base surgery (5 males and 6 females), with a mean age at aneurysm diagnosis of 62 years. PA surgery had been performed between 6 months and 25 years before aneurysm diagnosis. Four of these 11 patients also had multiple IAs. Previous brain radiotherapy was reported in 9 patients (12%) (4 males and 5 females), with a mean age at aneurysm diagnosis of 61 years. Radiotherapy had been performed 10 to 24 years before IA diagnosis; 6 of these patients had also undergone previous cranial base surgery. All patients who had undergone prior surgery and/or radiotherapy were diagnosed with a macroadenoma.

### Apoplexy

Pituitary apoplexy was observed in 9% of patients (*n* = 7), the majority of whom were male (*n* = 6). The mean age at IA diagnosis in this subgroup was 55 years. All patients had a macroadenoma; 4 had NF PAs and 3 had PRL PAs.

### Group 2 evaluation

**Fig. 2:**
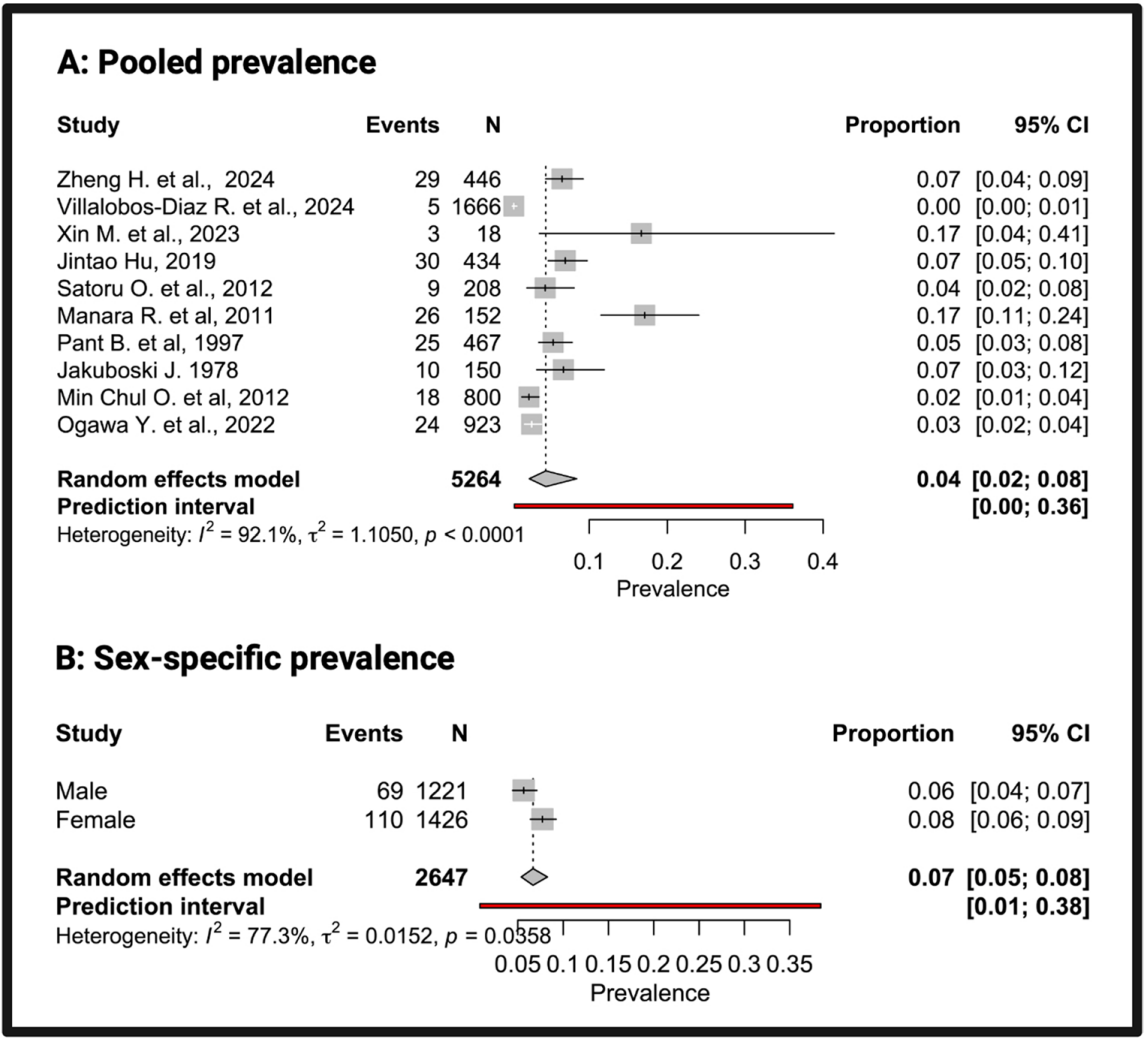
Title: Forest plot showing pooled (**A**) and sex-specific prevalence (**B**) of IAs in patients with PA Caption: A: Each square represents the prevalence estimate from an individual study, with size proportional to study weight, and horizontal lines indicating 95% confidence intervals (CI). The diamond represents the pooled prevalence calculated using a random-effects model. The red line indicates the prediction interval (0.00-0.36), reflecting between-study variability. B: Forest plot comparing prevalence between males and females

Data from 10 studies were included in the prevalence meta-analysis, with 5264 patients. Each study reported the number of patients with both PAs and IAs, along with the total population of patients with PAs. For each study, prevalence was calculated as the proportion of patients with concomitant PAs and IAs relative to the total number of patients with PAs, expressed as a percentage. Using a random-effects model, the pooled prevalence of IAs among patients with PAs was 4% (95% CI: 2%-4%) (Fig. 2A). A high degree of between-study heterogeneity was observed (I² = 92.1%, τ² = 1.1050, *p* < 0.0001). Where available, sex-specific prevalence was calculated (Fig. 2B), showing 8% (95% CI: 6%-9%) in females and 6% (95% CI: 4%-7%) in males. The overall prevalence was not significantly higher than the population proportion (*p* = 0.2043). In contrast, sex-specific analyses showed proportions significantly greater than the corresponding population proportions for both males (*p* < 0.01) and females (*p* < 0.01). The prevalence of multiple IAs ranged from 0 to 38% across studies (Supplementary Fig. 2). The pooled prevalence was 18%. Between-study heterogeneity was low (I² = 12.8%, τ² = 0.2089), and Cochran’s Q test was not statistically significant (*p* = 0.3274), indicating good consistency among studies.

## Discussion

Among adults without relevant comorbidities, IAs have an estimated prevalence of 3.2%, with sex-specific rates of ~ 1.5% in males and 6% in females [78–80]. In our meta-analysis, IA overall prevalence in patients with PA was 4%, slightly higher than that reported in the general population. However, sex-stratified analyses showed a prevalence of 6% in males and 8% in females, both significantly exceeding population estimates (*p* < 0.01), suggesting a possible sex-related difference in IA risk among PA patients. Such comparisons should be interpreted with caution, due to the lack of prospective studies.

In Group 1, females were more frequently affected than males, a distribution that mirrors findings reported both in cohorts of patients with PAs and in the general population [62, 78–80]. In contrast, Hu et al. found no statistically significant association between sex and IA prevalence in their PA cohort [70]. Overall, epidemiological data in the general population indicate a higher incidence of IAs in women compared to men, a difference that becomes especially evident after the fifth decade of life [78–80].

Our data show that NF PAs were the most frequent (40.5%), followed by GH PAs (31.1%) and PRL PAs (24.3%), in keeping with previous studies [66, 70]. In addition, most PAs in our cohort were macroadenomas, in line with previous reports [62, 66, 69]. Several mechanisms have been proposed to explain the increased IAs prevalence in patients with PAs. It has been hypothesized that invasive tumors may affect adjacent vessels through altered local hemodynamics, direct mechanical compression, or arterial wall infiltration [23, 30, 62, 70]. Supporting this notion, cavernous sinus invasion appears to be the tumor-related factor most consistently associated with IAs in patients with PAs [62, 69, 70]. Increased wall tension and augmented blood flow in tumor-feeding arteries have been suggested as contributing factors to aneurysm development in the perisellar region [23, 30, 62, 70]. Our data provide further support for this hypothesis, showing that IAs are more frequently located in the cavernous sinus segment of internal carotid artery (CS-ICA). Nevertheless, the precise mechanisms linking tumor invasiveness to aneurysm formation remain unclear [68, 70]. Beyond these local hemodynamic factors, systemic hormonal influences associated with specific PA types may also contribute to aneurysm development. Prolonged hypersecretion of GH has been proposed as a potential contributor to IAs development [48, 67, 81]. In vivo animal studies have shown that GH can alter type I/type III collagen ratio in arterial walls, resulting in increased arterial stiffness [82]. In a patient with acromegaly, similar changes in collagen composition, characterized by increased turnover and a relative deficiency of type III collagen have been observed in intracranial vessels, suggesting that these secondary connective tissue alterations may predispose individuals with GH-secreting PAs to IAs formation [48]. Although hypertension, diabetes, and dyslipidemia are common comorbidities in acromegaly due to elevated GH and IGF-1 levels, they have not been significantly associated with IAs. This observation suggests that the higher IAs prevalence in acromegalic patients is likely independent of GH/IGF-1-related metabolic factors [67]. Hyperprolactinemia has been associated with endothelial dysfunction and a pro-atherogenic vascular profile. Experimental and clinical data indicate that elevated prolactin levels may promote early vascular remodeling, including increased carotid intima–media thickness, through mechanisms involving insulin resistance, low-grade inflammation, and endothelial dysfunction [83]. Patients with prolactinomas show biochemical evidence of a pro-atherothrombotic state, characterized by increased endogenous thrombin and prothrombin levels, reduced HDL-cholesterol, and microvascular dysfunction [84].

Regarding IAs site, data from Group 1 show that the most frequent sites were CS-ICA (33%), followed by anterior communicating artery (ACoA) (17%). These findings partially mirror the distribution observed in the general population, where 37.2% of aneurysms are along the internal carotid artery, 18.7% originate from the ACoA [79, 80]. Moreover, multiple IAs were observed in 13.5% of patients in Group 1 and 18% in Group 2, rates that are comparable to those reported in the general population (15–30%) [85]. However, these comparisons should be interpreted as descriptive only.

Age appears to be an important determinant of IA occurrence in patients with PAs. Several studies suggest increased risk after midlife, although proposed thresholds vary across cohorts. Hu et al. identified a higher prevalence in patients older than 55 years [70], whereas Huang et al. reported a similar association from 50 years onward [62]. In our cohort, the highest prevalence was observed in patients aged 50–59 years, supporting a potential age-related contribution to vascular vulnerability.

Both in the general population and among patients with PAs, several risk factors have been implicated in IAs development. In the general population, the most relevant modifiable risk factors are arterial hypertension and cigarette smoking, which act synergistically to substantially increase aneurysm risk [79, 80]. We sought to investigate the contribution of these risk factors within Group 1. However, data availability was limited. Overall, both hypertension and smoking were markedly underreported across most studies included in the review, limiting the ability to draw definitive conclusions regarding their contribution to IA development in patients with PAs. In addition, it is important to consider additional factors that have been associated with IAs development in the general population: family history of IAs or subarachnoid hemorrhage suggests a significant genetic predisposition [79, 90]. Several genetic conditions, including autosomal dominant polycystic kidney disease, Ehlers-Danlos syndrome and Moyamoya disease, chronic inflammatory states, such as viral infections (e.g., uncontrolled HIV infection), severe periodontitis [78–80], excessive alcohol or cocaine use should be considered [80, 85]. None of these factors were reported among the studies included in our review. Nevertheless, rare cases may illustrate additional mechanisms. Adachi et al. reported a MEN1 case suggesting that skull-base thickening and platybasia due to MEN1-related hyperparathyroidism may contribute to aneurysm formation by causing local mechanical stress and altered cerebral blood flow, though the exact mechanisms remain unclear [49].

Prior radiotherapy has been suggested as contributing factor to IAs development in patient with PA [18, 23, 54]. Although rare, radiation-induced damage to the intracranial arterial wall may lead to aneurysm formation or rupture, sometimes resulting in life-threatening hemorrhagic events [36]. The latency between irradiation and IAs detection is typically long, ranging from 12 to 21 years [1, 36], supporting the need for long-term vascular surveillance after radiotherapy [62]. Proposed mechanisms include obliteration of the vasa vasorum and accelerated fibrotic or arteriosclerotic changes of the arterial wall [36]. Notably, 9 patients in Group 1 had previously undergone brain radiotherapy.

Previous cranial base surgery for PAs may represent a potential risk factor for IAs development [23]. In keeping with this finding, our results indicate that in Group 1, 15% of patients had undergone cranial base surgery prior to the occurrence of IAs.

Pituitary apoplexy is an uncommon but potentially life-threatening syndrome caused by acute hemorrhage and/or infarction of the pituitary gland, usually occurring in a pre-existing PA. It complicates approximately 2–12% of PAs, particularly NF PAs, and typically affects males in the fifth to sixth decades of life [86–88], consistent with our findings.

**Fig. 3:**
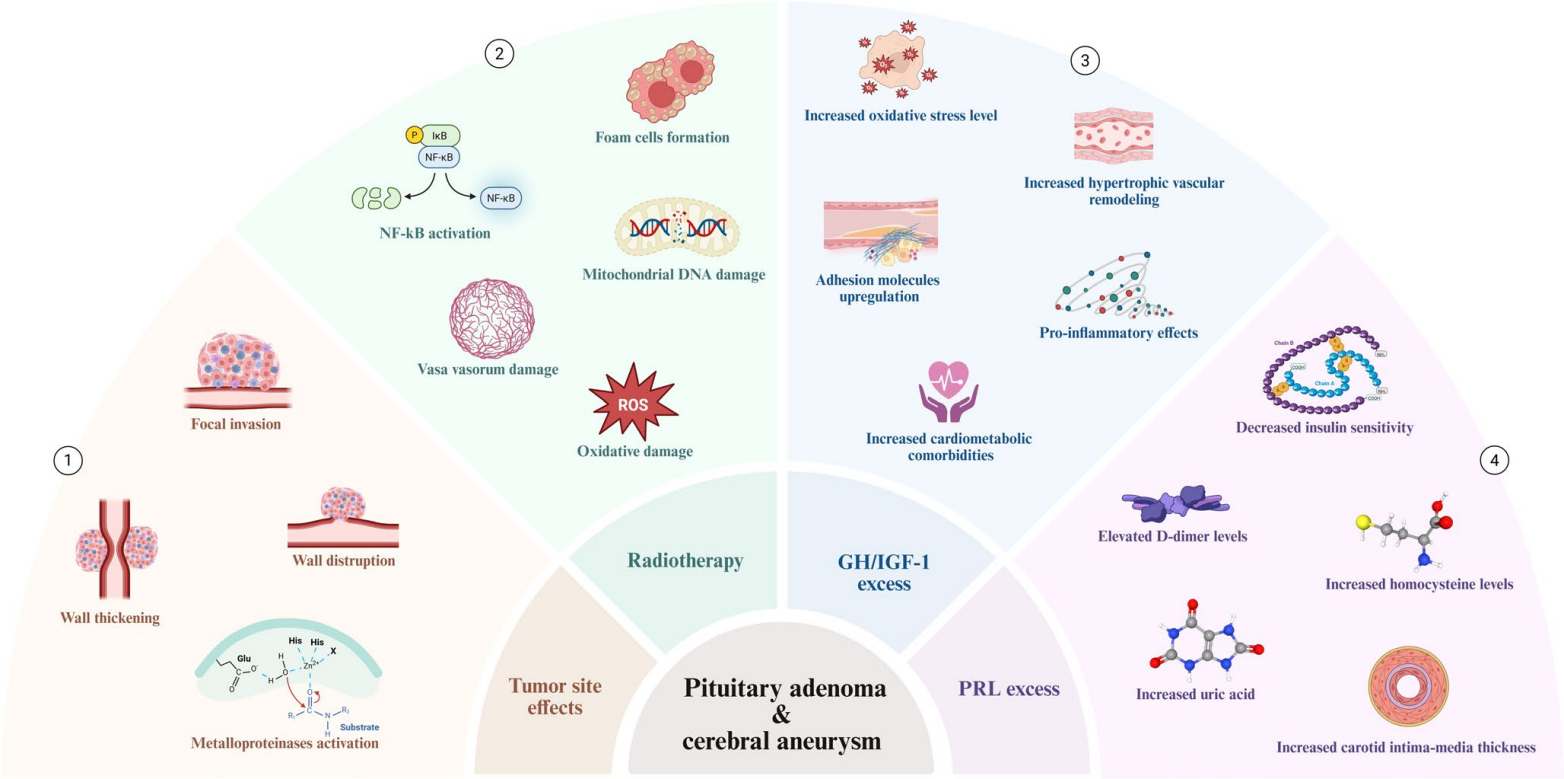
Title: Pathophysiological mechanisms underlying the coexistence of PA and IAs Caption: (1) Local effects of PA on adjacent arteries. PA invade the medial wall of the cavernous sinus with patterns including wall destruction, thickening, and focal infiltration. Invasive tumours overexpress metalloproteinases, which are also upregulated in early and progressive aneurysmal changes, supporting a shared mechanism of extracellular matrix degradation and vascular wall weakening. (2) Effects of radiotherapy. Radiotherapy causes endothelial loss and dysfunction via direct damage and ROS generation. Injury to the vasa vasorum may induce vascular wall ischemia. Oxidative stress and inflammation, partly mediated by NF-κB, promote lipid peroxidation and foam cell formation, initiating atherosclerosis. Preferential mitochondrial DNA damage, driven by calcium influx, amplifies ROS production, impairs the electron transport chain, and reduces nitric oxide (NO) availability. (3) Acromegaly-related vascular alterations. Acromegaly is characterized by increased oxidative stress, reduced antioxidant capacity, and decreased NO levels, leading to endothelial dysfunction. IGF-1 exerts pro-inflammatory effects, likely via MAPK signalling, and contributes to atherogenesis. GH/IGF-1 excess promotes endothelial adhesion molecule expression, inflammatory cell recruitment, and plaque formation, and is associated with cardiometabolic comorbidities that exacerbate vascular damage. Impaired endothelial progenitor cell function limits vascular repair, while vascular smooth muscle proliferation induces hypertrophic remodelling with increased wall thickness and wall-to-lumen ratio. (4) Hyperprolactinemia-related vascular effects. Hyperprolactinemia is associated with elevated inflammatory markers, endothelial dysfunction, and reduced insulin sensitivity. Increased homocysteine, D-dimer, and apoB/apoA-I ratio promote atherosclerosis and thrombogenicity. Consistently, increased carotid intima–media thickness supports the presence of subclinical vascular disease. Created in https://BioRender.com

Additional information concerning the possible mechanisms underlying the association between IAs and PAs are shown in Fig. 3 [89–103].

This meta-analysis has several limitations. Most of the available evidence derives from case reports, case series, and observational, often single-center or retrospective studies, which limits the overall generalizability of the findings. In many studies, the variables of interest were not primary outcomes, potentially leading to underreporting or misclassification. Importantly, substantial between-study heterogeneity was observed across analyses, which further reduces the precision and interpretability of pooled estimates. This heterogeneity likely reflects differences in study populations, methodological approaches, and diagnostic criteria. Moreover, the small number of included studies limit exploration of heterogeneity through subgroup analyses or meta-regression, further constraining the ability to identify its underlying sources. Finally, the small sample size of several included studies may hamper the robustness of the pooled estimates.

## Conclusion

We found that the overall IAs prevalence in patients with PAs is slightly higher than in the general population. Sex-stratified analyses suggest a significantly increased risk in both males and females. However, the high heterogeneity of the available data does not support routine universal screening for IAs in all patients with PAs. A more individualized, risk-adapted approach appears more appropriate. Vascular imaging may be considered in selected higher-risk patients, including those with macroadenomas, age over 50 years, and a history of prior cranial surgery or radiotherapy. In addition, patients with GH- or PRL-secreting macroadenomas may benefit from closer evaluation, given the potential vascular effects of hormonal hypersecretion. Prospective studies are needed to clarify pathophysiological mechanisms and to define evidence-based screening strategies.

## Supplementary Information

Below is the link to the electronic supplementary material.

Supplementary Material 1

Supplementary Material 2

Supplementary Material 3

Supplementary Material 4

Supplementary Material 5

Supplementary Material 6

Supplementary Material 7

Supplementary Material 8

## Data Availability

All data extracted and analyzed during this study are included in the main manuscript. These data are sufficient to allow replication of the presented analyses.
