# Supplementary material for: Pituitary adenoma and intracerebral aneurysms: case series, systematic review and meta-analysis
Source: Pituitary. 2026 May 16;29(3):85. doi: 10.1007/s11102-026-01690-w (PMC13179920; doi:10.1007/s11102-026-01690-w)
Supplement: Supplementary file 1 — Supplementary Material 1 [file 11102_2026_1690_MOESM1_ESM.pdf]

Pituitary

Pituitary neuroendocrine tumors and intracerebral aneurysms: systematic review and meta-analysis with a case series

Valentino Marino Picciola[1], Michela Borghesi[2], Vanessa Trombin[3], Serena Chirico[1], Maria Rosaria Ambrosio[1-3], Maria Chiara Zatelli[1-3]

Affiliations

[1]Section of Endocrinology, Geriatrics and Internal Medicine, Department of Medical Sciences, University of Ferrara, 44124 Ferrara, ITALY

[2]Department of Economics and Management, University of Ferrara

[3]Endocrine Unit, University Hospital S. Anna, 44124 Ferrara, ITALY

Corresponding Author

Prof. Maria Chiara Zatelli
Section of Endocrinology, Geriatrics and Internal Medicine
Department of Medical Sciences
University of Ferrara
Via Ariosto 35, 44100 - Ferrara
Phone: +39 0532 236682
Fax: +39 0532 236514
E-mail: ztlmch@unife.it

ORCID:

Valentino Marino Picciola: 0009-0005-5687-2208
Michela Borghesi: 0000-0003-1872-5766
Vanessa Trombin: 0009-0005-4674-1669
Serena Chirico: 0009-0006-9659-3374
Maria Rosaria Ambrosio: 0000-0002-7911-9770
Maria Chiara Zatelli: 0000-0001-8408-7796

Supplementary Figure 1: Distribution of IAs sites

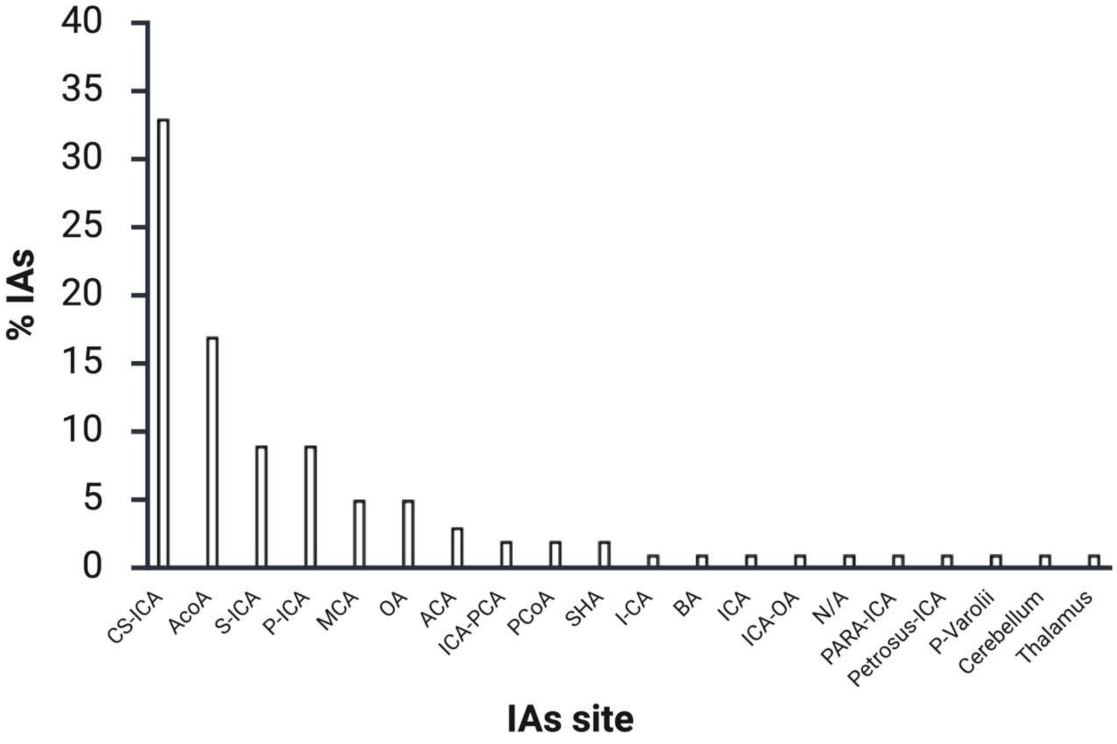

ICA = internal carotid artery; CS-ICA = cavernous sinus; ACoA = anterior communicating artery; S-ICA = supraclinoid ICA; P-ICA = paraclinoid ICA; MCA = middle cerebral artery; OA = ophthalmic artery; ACA = anterior cerebral artery; PCA = posterior cerebral artery; PCoA = posterior communicating artery; SHA = superior hypophyseal artery; I-ICA = infraclinoid ICA; BA = basilar artery; N/A = not available; PARA-ICA = paraophthalmic region; Petrosus-ICA; P-Varolii = Pons Varolii. Created in https://BioRender.com
