# Supplementary material for: Pituitary adenoma and intracerebral aneurysms: case series, systematic review and meta-analysis
Source: Pituitary. 2026 May 16;29(3):85. doi: 10.1007/s11102-026-01690-w (PMC13179920; doi:10.1007/s11102-026-01690-w)
Supplement: Supplementary file 3 — Supplementary Material 3 [file 11102_2026_1690_MOESM3_ESM.pdf]

Pituitary

# Pituitary neuroendocrine tumors and intracerebral aneurysms: systematic review and meta-analysis with a case series


Valentino Marino Picciola[1], Michela Borghesi[2], Vanessa Trombin[3], Serena Chirico[1], Maria Rosaria Ambrosio[1-3], Maria Chiara Zatelli[1-3]

**Affiliations**

[1]Section of Endocrinology, Geriatrics and Internal Medicine, Department of Medical Sciences, University of Ferrara, 44124 Ferrara, ITALY

[2]Department of Economics and Management, University of Ferrara

[3]Endocrine Unit, University Hospital S. Anna, 44124 Ferrara, ITALY

**Corresponding Author**

Prof. Maria Chiara Zatelli
Section of Endocrinology, Geriatrics and Internal Medicine
Department of Medical Sciences
University of Ferrara
Via Ariosto 35, 44100 - Ferrara
Phone: +39 0532 236682
Fax: +39 0532 236514
E-mail: ztlmch@unife.it

**ORCID**:

Valentino Marino Picciola: 0009-0005-5687-2208
Michela Borghesi: 0000-0003-1872-5766
Vanessa Trombin: 0009-0005-4674-1669
Serena Chirico: 0009-0006-9659-3374
Maria Rosaria Ambrosio: 0000-0002-7911-9770
Maria Chiara Zatelli: 0000-0001-8408-7796

**Supplementary Table 2**: Extracted Variables for Group 1 and Group 2

| | **Extracted Variables for Group 1** |
|---|---|
| **Bibliographic information** | First author, year of publication |
| **Patient demographics** | Age, sex |
| **IAs features** | Number per patient, age at diagnosis, anatomical site |
| **PAs features** | Size, hormonal function |
| **Apoplexy** | Presence of apoplexy |
| **Risk factors for IAs** | Radiotherapy, skull base surgery, hypertension, predisposing genetic diseases |
| | **Extracted Variables for Group 2** |
| **Population features of patients with PAs** | Total number, sex |
| **Features of patients with both IA and PA** | Number, sex, number of patients with multiple IAs |
