# Supplementary material for: Pituitary adenoma and intracerebral aneurysms: case series, systematic review and meta-analysis
Source: Pituitary. 2026 May 16;29(3):85. doi: 10.1007/s11102-026-01690-w (PMC13179920; doi:10.1007/s11102-026-01690-w)
Supplement: Supplementary file 4 — Supplementary Material 4 [file 11102_2026_1690_MOESM4_ESM.pdf]

Pituitary

Pituitary neuroendocrine tumors and intracerebral aneurysms: systematic review and meta-analysis with a case series

Valentino Marino Picciola[1], Michela Borghesi[2], Vanessa Trombin[3], Serena Chirico[1], Maria Rosaria Ambrosio[1-3], Maria Chiara Zatelli[1-3]

Affiliations

[1]Section of Endocrinology, Geriatrics and Internal Medicine, Department of Medical Sciences, University of Ferrara, 44124 Ferrara, ITALY

[2]Department of Economics and Management, University of Ferrara

[3]Endocrine Unit, University Hospital S. Anna, 44124 Ferrara, ITALY

Corresponding Author

Prof. Maria Chiara Zatelli
Section of Endocrinology, Geriatrics and Internal Medicine
Department of Medical Sciences
University of Ferrara
Via Ariosto 35, 44100 - Ferrara
Phone: +39 0532 236682
Fax: +39 0532 236514
E-mail: ztlmch@unife.it

ORCID:

Valentino Marino Picciola: 0009-0005-5687-2208
Michela Borghesi: 0000-0003-1872-5766
Vanessa Trombin: 0009-0005-4674-1669
Serena Chirico: 0009-0006-9659-3374
Maria Rosaria Ambrosio: 0000-0002-7911-9770
Maria Chiara Zatelli: 0000-0001-8408-7796

Supplementary Table 1: Search strategy across databases

Database	Search strategy
PubMed	(<br>"pituitary adenoma"[Mesh]<br>OR "pituitary adenoma*"[tiab]<br>OR "pituitary neoplasms"[Mesh]<br>OR "pituitary neoplasm*"[tiab]<br>OR "pituitary tumor*"[tiab]<br>OR "pituitary tumour*"[tiab]<br>OR "hypophyseal adenoma*"[tiab]<br>OR "sellar tumor*"[tiab]<br>OR "sellar tumour*"[tiab]<br>OR "pituitary neuroendocrine tumor"[tiab]<br>OR "pituitary neuroendocrine tumors"[tiab]<br>OR prolactinoma*[tiab]<br>OR "Prolactinoma"[Mesh]<br>OR acromegal*[tiab]<br>OR "Acromegaly"[Mesh]<br>OR "Cushing Disease"[Mesh]<br>OR "Cushing disease"[tiab]<br>OR gonadotropinoma*[tiab]<br>OR "growth hormone secreting adenoma*"[tiab]<br>OR "ACTH secreting adenoma*"[tiab]<br>OR "FSH secreting adenoma*"[tiab]<br>OR "LH secreting adenoma*"[tiab]<br>)<br>AND<br>(<br>"Intracranial Aneurysm"[Mesh]<br>OR aneurysm*[tiab]<br>OR "cerebral aneurysm*"[tiab]<br>OR "intracranial aneurysm*"[tiab]<br>OR "brain aneurysm*"[tiab]<br>OR "cerebrovascular aneurysm*"[tiab]<br>)<br>AND<br>("0001/01/01"[Date - Publication] : "2025/12/15"[Date - Publication])<br>AND (english[lang] OR italian[lang])
Embase	(<br>'pituitary adenoma'/exp<br>OR 'pituitary adenoma*':ti,ab<br>OR 'pituitary neoplasm'/exp<br>OR 'pituitary neoplasm*':ti,ab<br>OR 'pituitary tumor*':ti,ab<br>OR 'pituitary tumour*':ti,ab<br>OR 'hypophyseal adenoma*':ti,ab<br>OR 'sellar tumor*':ti,ab<br>OR 'sellar tumour*':ti,ab<br>OR 'pituitary neuroendocrine tumor':ti,ab<br>OR 'pituitary neuroendocrine tumors':ti,ab

	OR prolactinoma*:ti,ab<br>OR 'prolactinoma'/exp<br>OR acromegal*:ti,ab<br>OR 'acromegaly'/exp<br>OR 'cushing disease'/exp<br>OR 'cushing disease':ti,ab<br>OR gonadotropinoma*:ti,ab<br>OR 'growth hormone secreting adenoma*':ti,ab<br>OR 'acth secreting adenoma*':ti,ab<br>OR 'fsh secreting adenoma*':ti,ab<br>OR 'lh secreting adenoma*':ti,ab<br>)<br>AND<br>(<br>'intracranial aneurysm'/exp<br>OR aneurysm*:ti,ab<br>OR 'cerebral aneurysm*':ti,ab<br>OR 'intracranial aneurysm*':ti,ab<br>OR 'brain aneurysm*':ti,ab<br>OR 'cerebrovascular aneurysm*':ti,ab<br>)<br>AND [<1966-2025]/py<br>AND ([english]/lim OR [italian]/lim)
