# Supplementary material for: Pituitary adenoma and intracerebral aneurysms: case series, systematic review and meta-analysis
Source: Pituitary. 2026 May 16;29(3):85. doi: 10.1007/s11102-026-01690-w (PMC13179920; doi:10.1007/s11102-026-01690-w)
Supplement: Supplementary file 5 — Supplementary Material 5 [file 11102_2026_1690_MOESM5_ESM.pdf]

Pituitary

Pituitary neuroendocrine tumors and intracerebral aneurysms: systematic review and meta-analysis with a case series

Valentino Marino Picciola[1], Michela Borghesi[2], Vanessa Trombin[3], Serena Chirico[1], Maria Rosaria Ambrosio[1-3], Maria Chiara Zatelli[1-3]

Affiliations

[1]Section of Endocrinology, Geriatrics and Internal Medicine, Department of Medical Sciences, University of Ferrara, 44124 Ferrara, ITALY

[2]Department of Economics and Management, University of Ferrara

[3]Endocrine Unit, University Hospital S. Anna, 44124 Ferrara, ITALY

Corresponding Author

Prof. Maria Chiara Zatelli

Section of Endocrinology, Geriatrics and Internal Medicine

Department of Medical Sciences

University of Ferrara

Via Ariosto 35, 44100 - Ferrara

Phone: +39 0532 236682

Fax: +39 0532 236514

E-mail: ztlmch@unife.it

ORCID:

Valentino Marino Picciola: 0009-0005-5687-2208

Michela Borghesi: 0000-0003-1872-5766

Vanessa Trombin: 0009-0005-4674-1669

Serena Chirico: 0009-0006-9659-3374

Maria Rosaria Ambrosio: 0000-0002-7911-9770

Maria Chiara Zatelli: 0000-0001-8408-7796

Supplementary Table 5: Results of risk of bias assessment for Group 2 based on Joanna Briggs Institute Checklist for Prevalence Studies

Study, year	Q1	Q2	Q3	Q4	Q5	Q6	Q7	Q8	Q9	Score	Quality
Jakuboski J. 1978	Yes	Yes	Yes	Yes	Yes	Yes	Yes	Yes	NA	8	High
Jintao Hu. et al., 2023	Yes	Yes	Yes	Yes	Yes	Yes	Yes	Yes	NA	8	High
Manara R. et al, 2011	Yes	Yes	Yes	Yes	Yes	Yes	Yes	Yes	NA	8	High
Min Chul O. et al, 2012	Yes	Yes	Yes	Yes	Yes	Yes	Yes	Yes	NA	8	High
Ogawa Y. et al., 2022	Yes	Yes	Yes	No	Yes	Yes	Yes	Yes	NA	7	Moderate
Pant B. et al, 1997	Yes	Yes	Yes	Yes	Yes	Unc	Unc	Yes	NA	6	Moderate
Satoru O. et al., 2012	Yes	Yes	Yes	Yes	Yes	Yes	Yes	Yes	NA	8	High
Villalobos-Diaz R. et al., 2024	Yes	Yes	Yes	No	Yes	Unc	Unc	Yes	NA	5	Low
Xin M. et al., 2023	Yes	Yes	Yes	No	Yes	Yes	Yes	Yes	NA	7	Moderate
Zheng H. et al., 2024	Yes	Yes	Yes	No	Yes	Yes	Yes	Yes	NA	7	Moderate

The Joanna Briggs Institute Checklist for prevalence studies includes nine questions (Q): Q1 = Was the sample frame appropriate to address the target population?; Q2 = Were study participants sampled in an appropriate way?; Q3 = Was the sample size adequate?; Q4 = Were the study subjects and the setting described in detail?; Q5 = Was the data analysis conducted with sufficient coverage of the identified sample?; Q6 = Were valid methods used for the identification of the condition?; Q7 = Was the condition measured in a standard, reliable way for all participants?; Q8 = Was there appropriate statistical analysis?; Q9 = Was the response rate adequate, and if not, was the low response rate managed appropriately? Each item for both tools was rated as “Yes”, “No”, “Unclear”, or “Not Applicable” by two independent reviewers, with disagreements resolved by consensus. Overall study quality was determined based on the total number of “Yes” responses. Studies were classified as low quality (0–5), moderate quality (6–7), or high quality (8–9). Unc = Unclear; NA = Not Applicable. Villalobos-Diaz R. et al. (2024) was assessed twice for risk of bias due to the presence of two distinct components: prevalence data and a case report the meet inclusion criteria for Group 1. This approach was adopted to ensure a more accurate and design-specific evaluation of methodological quality.
