# Supplementary material for: Pituitary adenoma and intracerebral aneurysms: case series, systematic review and meta-analysis
Source: Pituitary. 2026 May 16;29(3):85. doi: 10.1007/s11102-026-01690-w (PMC13179920; doi:10.1007/s11102-026-01690-w)
Supplement: Supplementary file 6 — Supplementary Material 6 [file 11102_2026_1690_MOESM6_ESM.pdf]

Pituitary

Pituitary neuroendocrine tumors and intracerebral aneurysms: systematic review and meta-analysis with a case series

Valentino Marino Picciola[1], Michela Borghesi[2], Vanessa Trombin[3], Serena Chirico[1], Maria Rosaria Ambrosio[1-3], Maria Chiara Zatelli[1-3]

Affiliations

[1]Section of Endocrinology, Geriatrics and Internal Medicine, Department of Medical Sciences, University of Ferrara, 44124 Ferrara, ITALY

[2]Department of Economics and Management, University of Ferrara

[3]Endocrine Unit, University Hospital S. Anna, 44124 Ferrara, ITALY

Corresponding Author

Prof. Maria Chiara Zatelli

Section of Endocrinology, Geriatrics and Internal Medicine

Department of Medical Sciences

University of Ferrara

Via Ariosto 35, 44100 - Ferrara

Phone: +39 0532 236682

Fax: +39 0532 236514

E-mail: ztlmch@unife.it

ORCID:

Valentino Marino Picciola: 0009-0005-5687-2208

Michela Borghesi: 0000-0003-1872-5766

Vanessa Trombin: 0009-0005-4674-1669

Serena Chirico: 0009-0006-9659-3374

Maria Rosaria Ambrosio: 0000-0002-7911-9770

Maria Chiara Zatelli: 0000-0001-8408-7796

Supplementary Table 4: Results of risk of bias assessment for Group 1 based on Joanna Briggs Institute Checklist for Case Series

Author, year	Q1	Q2	Q3	Q4	Q5	Q6	Q7	Q8	Q9	Q10	Score	Quality
Acqui M. et al., 1987	Unc	No	Yes	Unc	No	Yes	Yes	Yes	Yes	Yes	6	Low
Fujiwara S. et al., 1991	Yes	Yes	Yes	Unc	Yes	Yes	Yes	Yes	Yes	NA	8	Medium
Wakai S. et al., 1979	Yes	Yes	Yes	Yes	Unc	Yes	Yes	Yes	Yes	Yes	9	High
Yamashita S. et al., 2023	Yes	Yes	Yes	Unc	No	Yes	Yes	Yes	Yes	NA	7	Medium

The Joanna Briggs Institute Checklist for Case Reports includes eight questions (Q): Q1 = Were there clear criteria for inclusion in the case series?; Q2 = Was the condition measured in a standard, reliable way for all participants included in the case series?; Q3 = Were valid methods used for identification of the condition for all participants included in the case series?; Q4 = Did the case series have consecutive inclusion of participants?; Q5 = Did the case series have complete inclusion of participants?; Q6 = Was there clear reporting of the demographics of the participants in the study?; Q7 = Was there clear reporting of clinical information of the participants?; Q8 = Were the outcomes or follow up results of cases clearly reported?; Q9 = Was there clear reporting of the presenting site(s)/clinic(s) demographic information?; Q10 = Was statistical analysis appropriate?

Each item for both tools was rated as “Yes”, “No”, “Unclear”, or “Not Applicable” by two independent reviewers, with disagreements resolved by consensus. Overall study quality was determined based on the total number of “Yes” responses. Studies were classified as low quality (0–6), moderate quality (7-8), or high quality (9–10). Unc = Unclear; NA = Not Applicable.
