# Supplementary material for: Pituitary adenoma and intracerebral aneurysms: case series, systematic review and meta-analysis
Source: Pituitary. 2026 May 16;29(3):85. doi: 10.1007/s11102-026-01690-w (PMC13179920; doi:10.1007/s11102-026-01690-w)
Supplement: Supplementary file 7 — Supplementary Material 7 [file 11102_2026_1690_MOESM7_ESM.pdf]

Pituitary

Pituitary neuroendocrine tumors and intracerebral aneurysms: systematic review and meta-analysis with a case series

Valentino Marino Picciola[1], Michela Borghesi[2], Vanessa Trombin[3], Serena Chirico[1], Maria Rosaria Ambrosio[1-3], Maria Chiara Zatelli[1-3]

Affiliations

[1]Section of Endocrinology, Geriatrics and Internal Medicine, Department of Medical Sciences, University of Ferrara, 44124 Ferrara, ITALY

[2]Department of Economics and Management, University of Ferrara

[3]Endocrine Unit, University Hospital S. Anna, 44124 Ferrara, ITALY

Corresponding Author

Prof. Maria Chiara Zatelli

Section of Endocrinology, Geriatrics and Internal Medicine

Department of Medical Sciences

University of Ferrara

Via Ariosto 35, 44100 - Ferrara

Phone: +39 0532 236682

Fax: +39 0532 236514

E-mail: ztlmch@unife.it

ORCID:

Valentino Marino Picciola: 0009-0005-5687-2208

Michela Borghesi: 0000-0003-1872-5766

Vanessa Trombin: 0009-0005-4674-1669

Serena Chirico: 0009-0006-9659-3374

Maria Rosaria Ambrosio: 0000-0002-7911-9770

Maria Chiara Zatelli: 0000-0001-8408-7796

Supplementary Table 3: Results of risk of bias assessment for Group 1 based on Joanna Briggs Institute Checklist for Case Reports

**Author, year**	**Q1**	**Q2**	**Q3**	**Q4**	**Q5**	**Q6**	**Q7**	**Q8**	**Score**	**Quality**
Adachi K. et al., 1993	Yes	No	Yes	Yes	N/A	N/A	No	No	3	Low
Agarwal H. et al., 2018	Yes	No	No	Yes	Yes	Yes	Yes	No	5	Medium
Akutsu N. et al., 2014	Yes	No	Unclear	Yes	Yes	Yes	No	Unclear	4	Medium
Almeida Silva J.M. et al., 2013	Yes	Yes	Yes	Unclear	Yes	Yes	Yes	Yes	7	High
Bulsara K. et al., 2007	Yes	No	Yes	Yes	Yes	Yes	Yes	Yes	7	High
Choi H.S. et al., 2013	Yes	Unclear	Unclear	Yes	Yes	Yes	Yes	Yes	6	High
Chuang C.C. et al., 2006	Yes	No	Yes	Yes	Yes	Yes	Yes	Unclear	6	High
Cifuentes-Lobelo H. et al., 2024	Yes	Yes	Yes	Yes	Yes	Yes	Yes	Yes	8	High
Curto L. et al., 2007	Yes	Yes	Yes	Yes	Yes	Yes	Yes	Yes	8	High
Fedder S.L. et al., 1990	Yes	Yes	Unclear	Unclear	Yes	No	Yes	Yes	5	Medium
Fookeerah P. & McLean M., 2023	Yes	Yes	Yes	Yes	Yes	Yes	No	Unclear	6	High
Giammusso V. et al., 1960	Yes	Yes	Yes	Yes	Unclear	No	No	No	4	Medium
Gokalp H.Z. et al., 1980	Yes	No	Unclear	Yes	Unclear	Yes	Yes	No	4	Medium
Gu Y. et al., 2022	Yes	Unclear	Unclear	Yes	Yes	Yes	Yes	Yes	6	High
He W. et al., 2024	Yes	Yes	Unclear	Yes	Yes	Yes	Yes	Yes	7	High
Hermier M. et al., 1994	Yes	Yes	Yes	Yes	Yes	N/A	Yes	Yes	7	High
Hidenori E. et al., 2011	Yes	Yes	No	Yes	Yes	Yes	Yes	Yes	7	High
Holdaway M. et al., 2023	Yes	No	Unclear	Yes	Yes	Yes	Yes	Yes	6	High
Hori T. et al., 1982	Yes	Yes	Yes	Yes	Yes	Yes	Yes	Yes	8	High
Imamura J et al., 1998	Yes	Yes	Yes	Yes	N/A	N/A	No	Unclear	4	Medium
Inoue H. et al., 2019	Yes	Yes	No	Yes	Yes	N/A	Yes	Yes	6	High
Jordan R.M. et al., 1978	Yes	No	Yes	Yes	Yes	N/A	Yes	Yes	6	High
Keenan J.P. et al., 2021	Yes	No	No	Yes	Yes	Yes	Yes	Unclear	5	Medium
Khachatryan T. et al., 2018	Yes	Yes	Yes	Yes	Yes	Yes	Yes	Yes	8	High
Khalsa S.S. et al., 2016	Yes	Yes	No	Yes	Yes	Yes	Yes	No	6	High
Kino H. et al., 2020	Yes	Yes	No	Yes	Yes	Yes	No	Yes	6	High
Lippman H.H. et al., 1971	Yes	Yes	Yes	Yes	Yes	Yes	Yes	Yes	8	High
Mangiardi J. R. et al., 1983	Yes	Yes	Yes	Yes	N/A	N/A	Yes	Yes	6	High
McConachie N.S. & Jacobson I., 1994	Yes	Yes	Unclear	Yes	Unclear	Yes	Yes	No	5	Medium
Mondragon-Soto M.G. et al., 2022	Yes	Yes	Yes	Yes	Yes	Yes	Yes	Yes	8	High
Nadjem H. et al., 2007	Yes	Yes	Yes	Yes	Yes	Yes	No	Yes	7	High
Nakahara M. et al., 2018	Yes	No	Unclear	Yes	Yes	Yes	Yes	No	5	Medium
Nene A. et al., 2022	Yes	Unclear	Unclear	Yes	Yes	Yes	No	Yes	5	Medium
Nishijima Y. et al., 2010	Yes	No	Yes	Yes	Yes	Yes	Yes	Unclear	6	High
Pedrozo C.F. et al., 2020	Yes	Unclear	Yes	Yes	Yes	Yes	Yes	Unclear	6	High
Rennert R.C. et al., 2022	Yes	No	Yes	Unclear	Yes	Yes	Yes	No	5	Medium
Revuelta R. et al., 2002	Yes	Yes	Yes	Yes	Yes	Yes	Yes	Yes	8	High
Sade B. et al., 2004	Yes	No	Yes	Yes	Yes	Yes	Yes	No	6	High
Saito T. et al., 2017	Yes	Yes	Yes	Unclear	Yes	Yes	Yes	Unclear	6	High
Salpietro F.M. et al., 1997	Yes	Unclear	Unclear	Yes	Yes	Yes	Yes	No	5	Medium
Satyarthee G.D. & Raheja A., 2017	Yes	Yes	Yes	Yes	Yes	Yes	Yes	Unclear	7	High
Schenk V.W. & Solleveld H., 1968	Yes	Yes	Yes	Yes	N/A	N/A	No	Unclear	4	Medium
Seda L. Jr et al., 2008	Yes	Yes	Yes	Yes	Yes	Yes	Yes	Yes	8	High
Shahlaie K. et al., 2005	Yes	Yes	Yes	Yes	Yes	Yes	Yes	No	7	High

Soni A. et al., 2008	Yes	Yes	Yes	Yes	Yes	Yes	Yes	Yes	8	High
Taniguchi T. et al., 2021	Yes	Yes	Yes	Yes	Yes	Yes	No	Yes	7	High
Tian X. et al., 2015	Yes	Yes	Yes	Yes	Yes	Yes	Yes	No	7	High
Troisi F. et al., 1969	Yes	Yes	Yes	Yes	Yes	Yes	No	No	6	High
Villalobos-Diaz R. et al., 2024*	Yes	Yes	Yes	Yes	Yes	Yes	Yes	Unclear	7	High
Wang C.S. et al., 2009	Yes	Yes	Yes	Yes	Yes	Yes	Yes	No	7	High
Wang T. et al., 2021	Yes	Unclear	Unclear	Yes	Yes	Yes	Yes	No	5	Medium
Xia X. et al., 2011	Yes	Unclear	Yes	Yes	Yes	Yes	Yes	No	6	High
Xu K. et al., 2015	Yes	Unclear	Yes	Yes	Yes	Yes	Yes	Yes	7	High
Yamada S. et al., 2012	Yes	No	No	Yes	Yes	Yes	Yes	No	5	Medium
Yang M.Y. et al., 2005	Yes	Unclear	Unclear	Yes	Yes	Yes	Yes	Yes	6	High
Yoshida M. et al., 2021	Yes	No	Yes	Yes	Yes	Yes	Yes	Yes	7	High
Yu K. et al., 2011	Yes	No	Unclear	Yes	Yes	Yes	Yes	Yes	6	High
Zatelli M.C. et al., 2004	Yes	Yes	Unclear	Yes	Yes	Yes	Yes	Yes	7	High

The Joanna Briggs Institute Checklist for Case Reports includes eight questions (Q): Q1 = Were patient’s demographic characteristics clearly described?; Q2 = Was the patient’s history clearly described and presented as a timeline?; Q3 = Was the current clinical condition of the patient on presentation clearly described?; Q4 = Were diagnostic tests or assessment methods and the results clearly described?; Q5 = Was the intervention(s) or treatment procedure(s) clearly described?; Q6 = Was the post-intervention clinical condition clearly described?; Q7 = Were adverse events (harms) or unanticipated events identified and described?; Q8 = Does the case report provide takeaway lessons? Each item for both tools was rated as “Yes”, “No”, “Unclear”, or “Not Applicable” by two independent reviewers, with disagreements resolved by consensus. Overall study quality was determined based on the total number of “Yes” responses. Studies were classified as low quality (0–4), moderate quality (5–6), or high quality (7–8). NA = Not Applicable. Villalobos-Diaz R. et al. (2024) was assessed twice for risk of bias due to the presence of two distinct components: prevalence data and a case report the meet inclusion criteria for Group 1. This approach was adopted to ensure a more accurate and design-specific evaluation of methodological quality.
