# Supplementary material for: Pituitary adenoma and intracerebral aneurysms: case series, systematic review and meta-analysis
Source: Pituitary. 2026 May 16;29(3):85. doi: 10.1007/s11102-026-01690-w (PMC13179920; doi:10.1007/s11102-026-01690-w)
Supplement: Supplementary file 8 — Supplementary Material 8 [file 11102_2026_1690_MOESM8_ESM.pdf]

Pituitary

Pituitary neuroendocrine tumors and intracerebral aneurysms: systematic review and meta-analysis with a case series

Valentino Marino Picciola[1], Michela Borghesi[2], Vanessa Trombin[3], Serena Chirico[1], Maria Rosaria Ambrosio[1-3], Maria Chiara Zatelli[1-3]

Affiliations

[1]Section of Endocrinology, Geriatrics and Internal Medicine, Department of Medical Sciences, University of Ferrara, 44124 Ferrara, ITALY

[2]Department of Economics and Management, University of Ferrara

[3]Endocrine Unit, University Hospital S. Anna, 44124 Ferrara, ITALY

Corresponding Author

Prof. Maria Chiara Zatelli

Section of Endocrinology, Geriatrics and Internal Medicine

Department of Medical Sciences

University of Ferrara

Via Ariosto 35, 44100 - Ferrara

Phone: +39 0532 236682

Fax: +39 0532 236514

E-mail: ztlmch@unife.it

ORCID:

Valentino Marino Picciola: 0009-0005-5687-2208

Michela Borghesi: 0000-0003-1872-5766

Vanessa Trombin: 0009-0005-4674-1669

Serena Chirico: 0009-0006-9659-3374

Maria Rosaria Ambrosio: 0000-0002-7911-9770

Maria Chiara Zatelli: 0000-0001-8408-7796

Supplementary Table 6: R codes used for meta-analyses.

**R code used for pooled prevalence of IAs in patients with PA**
> library(meta)<br>> m = metaprop(event = Total.PA.and.IA, n = Total.PA, studlab = study, data = data, sm = "PLOGIT", method = "GLMM", random = TRUE, common = FALSE)<br>> forest(m, prediction = TRUE, xlab = "Prevalence", leftcols = c("studlab", "event", "n"), leftlabs = c("Study", "Events", "N"), rightcols = c("effect", "ci"), rightlabs = c("Proportion", "95% CI"))
**R code used for sex-specific prevalence of IAs in patients with PA**
> library(meta)<br>> cases = c(69, 110)<br>> total = c(1221, 1426)<br>> groups = c("Male", "Female")<br>> m = metaprop(event = cases, n = total, studlab = groups, sm = "PLOGIT", method = "GLMM", random = TRUE, common = FALSE)<br>> forest(m, prediction = TRUE, xlab = "Prevalence", leftcols = c("studlab", "event", "n"), leftlabs = c("Study", "Events", "N"), rightcols = c("effect", "ci"), rightlabs = c("Proportion", "95% CI"))
**R code used for prevalence of multiple IAs in patients with PA**
> library(meta)<br>> m = metaprop(event = Number.of.multiple.IA, n = Total.PA.and.IA, studlab = study, data = data, sm = "PLOGIT", method = "GLMM", random = TRUE, common = FALSE)<br>> forest(m, prediction = TRUE, xlab = "Prevalence", leftcols = c("studlab", "event", "n"), leftlabs = c("Study", "Events", "N"), rightcols = c("effect", "ci"), rightlabs = c("Proportion", "95% CI"))

Meta-analyses for both pooled and sex-specific prevalence were performed using the *metaprop* function from the *meta* package in R. Proportions were estimated using a generalized linear mixed model (GLMM) with logit link transformation under a random-effects framework, accounting for between-study heterogeneity and the binomial distribution of the data. Between-study variance was estimated using maximum likelihood methods.
